# Medical Department U. S. Navy

**Published:** 1852-12

**Authors:** 


### THE MEDICAL EXAMINER.

PHILADELPHIA, DECEMBER, 1852.

MEDICAL DEPARTMENT, U. S. NAVY.

A Board for the examination of Assistant Surgeons for promotion, and of candidates for admission, will convene at the Naval Asylum, Philadelphia, on 15th Dec. The Board will be composed as follows:

*Surgeon,* WM. P. C. BARTON, *President.*

*Surgeons,* CORNICK, DILLARD, HORNER and RUSCHENBERGER, Members.

